# Ponseti method in the management of clubfoot under 2 years of age: A systematic review

**DOI:** 10.1371/journal.pone.0178299

**Published:** 2017-06-20

**Authors:** Balasankar Ganesan, Ameersing Luximon, Adel Al-Jumaily, Suchita Kothe Balasankar, Ganesh R. Naik

**Affiliations:** 1 The Hong Kong Polytechnic University, Hung Hom, Hong Kong SAR; 2 Centre for Health Technology (CHT), Faculty of Engineering and IT, University of Technology Sydney (UTS), Ultimo, Sydney, Australia; 3 Maharashtra University of Health Sciences, Nasik, India; Harvard Medical School/BIDMC, UNITED STATES

## Abstract

**Background:**

Congenital talipes equinovarus (CTEV), also known as clubfoot, is common congenital orthopedic foot deformity in children characterized by four components of foot deformities: hindfoot equinus, hindfoot varus, midfoot cavus, and forefoot adduction. Although a number of conservative and surgical methods have been proposed to correct the clubfoot deformity, the relapses of the clubfoot are not uncommon. Several previous literatures discussed about the technical details of Ponseti method, adherence of Ponseti protocol among walking age or older children. However there is a necessity to investigate the relapse pattern, compliance of bracing, number of casts used in treatment and the percentages of surgical referral under two years of age for clear understanding and better practice to achieve successful outcome without or reduce relapse. Therefore this study aims to review the current evidence of Ponseti method (manipulation, casting, percutaneous Achilles tenotomy, and bracing) in the management of clubfoot under two years of age.

**Materials and methods:**

Articles were searched from 2000 to 2015, in the following databases to identify the effectiveness of Ponseti method treatment for clubfoot: Medline, Cumulative Index to Nursing and Allied Health Literature (CINHAL), PubMed, and Scopus. The database searches were limited to articles published in English, and articles were focused on the effectiveness of Ponseti method on children with less than 2 years of age.

**Results:**

Of the outcome of 1095 articles from four electronic databases, twelve articles were included in the review. Pirani scoring system, Dimeglio scoring system, measuring the range of motion and rate of relapses were used as outcome measures.

**Conclusions:**

In conclusion, all reviewed, 12 articles reported that Ponseti method is a very effective method to correct the clubfoot deformities. However, we noticed that relapses occur in nine studies, which is due to the non-adherence of bracing regime and other factors such as low income and social economic status.

## Introduction

Congenital talipes equinovarus (CTEV) or clubfoot is one of the most common pediatric foot deformity occurs at 1 in 1000 live births [1, 2]. It consists of four components: Ankle equinus, hindfoot varus, forefoot adductus, and midfoot cavus [3–6]. Although, there are a number of conservative or non-conservative treatments have been used to correct the clubfoot, it is still challenging to treat the most severe cases of clubfoot. For the last 150 years, the treatment methods used for clubfoot are still controversial [7]. Because, the extensive surgical procedures (repeated soft tissue releases) on the clubfoot lead to induce some complications such as stiffness of foot, arthritic problems and poor quality of life [8]. After that, a number of conservative methods are proposed to correct the clubfoot deformity with the following techniques such as different methods of manipulations, orthosis or splinting or bracing, casting, and strapping [9–12]. Historically, conservative management was introduced by Hippocrates in around 400 BC [13, 14]. Later, in 1939, Kite introduced his method [15], referred as Kite method, which is including manipulation and casting technique, but the success rate of this method was poor [7, 11, 16]. Subsequently, in 1963, Ponseti developed a conservative method, called as Ponseti method, with manipulation, casting, Achilles tenotomy and bracing, and it takes about four to five weeks to achieve the full correction of all four components of the clubfoot deformity [17, 18]. In this method, Achilles tenotomy is used to release the equinus deformity and bracing for maintaining the corrected clubfoot [19, 20], and it helps to obtain the plantigrade, functional, pain-free foot [21]. Although orthopedic surgeons agreed that initial treatment for clubfoot should be a conservative method to correct the clubfoot successfully [17, 22–27], the relapses, partial correction of clubfoot- rocker bottom foot is still not avoidable [28, 29]. Based on the literature search, in the past five decades, a number of studies have reviewed and published which include the history of development of conservative method and its management in the clubfoot [30, 31], controversies in the clubfoot management [32], current updates of clubfoot treatment and effectiveness of Ponseti method [1, 33, 34], different types of conservative methods (Ponseti techniques, Kite’s method, and French physical therapy method) and results of Ponseti methods [35], using sonography for the evaluation of clubfoot treatment outcome [36]. For a period of century, to the best of our knowledge, there were only 5 systematic review articles published on the clubfoot with related Ponseti management [37–41] and one as Cochrane review [42], in which Smythe, Kuper (41). study discussed about the birth prevalence of clubfoot in the group of low- and middle-income countries. Although other studies reviewed the relapses, bracing protocol, and percutaneous tenotomy, there were no studies systematically reviewed specifically in a particular age population especially children under the age of two years. Despite several studies reported that initial presentation, Ponseti techniques provide more successful results [17, 43, 44], the clubfoot relapses or recurrences of the clubfoot can be still seen in the children with less than 2 years of age followed by the Ponseti method of treatment. Adherence of Ponseti and his collogues protocol is necessary to achieve full successful clubfoot treatment without relapses or any deformities. Several literatures review articles reported the technical details and adherence of Ponseti protocol among walking age or older children. Initially, Ponseti developed his method and its protocol for children with clubfoot below the age of two years. However, most of the clubfoot based review studies focused either the group of children with walking age or over the age of 10 years [45], and hence the future studies should be focused with number of casts, percentages of surgical procedures, frequency and management of clubfoot relapses [46]. Therefore, this systematic review study aims to investigate the following details in the children with less than 2 years treated with Ponseti method: A) to review how the Ponseti treatment technical regime strictly followed in their study to achieve the initial correction B) to find the outcome of the study, including success rate, number of casts and percentages of surgical recommendations, and to review the relapses and relapse pattern of the clubfoot causative factors for relapses.

## Materials and methods

### Search strategy

**Fig 1:**
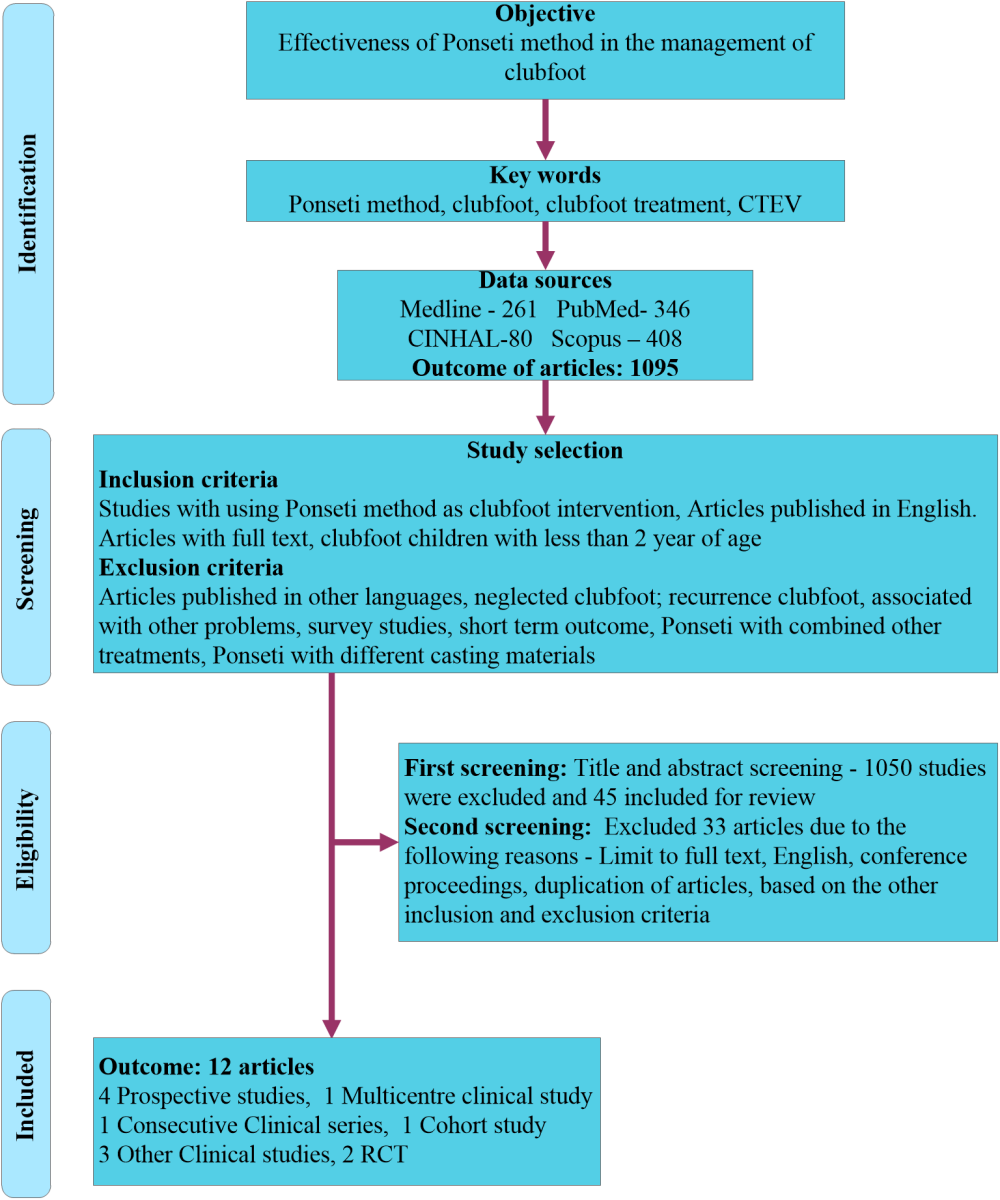
Flow chart of literature search and recruitment process.

A systematic literature search was performed by three authors (BG, GRN, and SB) for articles published between from January 2000 to September 2015 in the following electronic databases: Medline, Cumulative Index to Nursing and Allied Health Literature (CINHAL), PubMed, Scopus for relevant articles to identify the Ponseti method of treatment to treat clubfoot. The process of literature search and articles selected for this study is illustrated in Fig 1.The flow chart summarizes the method of the literature search, inclusion and exclusion criteria for selecting articles, method of extraction of the articles, outcome of the articles, and results. The following key words were used in the electronic databases: "Clubfoot or CTEV or congenital talipes equinovarus", "Ponseti method or Ponseti treatment'', "clubfoot treatment or clubfoot management" (“S1 File"). The articles published in the English language were only considered for this review. In addition, we followed the guidelines of Preferred Reporting Items for Systematic Reviews and Meta-Analyses (PRISMA) in this systematic review study (“S2 File").

### Study selection

The title and abstract of the Ponseti method for clubfoot related articles were reviewed and extracted by investigators (BG, AL, AA, and GRN) based on the inclusion criteria and exclusion criteria of this study. The following information was examined in the abstract and title for selecting the articles: **Inclusion criteria:** Articles were selected based on the following inclusion criteria: Studies with using the Ponseti method as an intervention for clubfoot deformities, articles restricted into the language of English with full text, clubfoot children with less than 2 years. According to the Ponseti classification, children with less than 2 years are considered as untreated clubfoot [47, 48]. **Exclusion criteria:** Articles published in other languages, neglected clubfoot, associated with other problems, survey studies, short-term outcome, surgical management of clubfoot and Ponseti method of intervention for testing the effectiveness of different casting materials were excluded in this systematic review.

### Data extraction

Two authors extracted data on characteristics of clubfoot patients from the selected articles and recorded into the data extraction sheet, followed by the extracted data was reviewed by two authors for accuracy. The following characteristics of clubfoot patients and selected articles were included into the data extraction sheet: study design, sample size and number of children, number of feet, types of intervention, intervention groups, gender, affected side of the clubfoot (bilateral & unilateral clubfoot), age at initial casting, number of casting, schedule of bracing protocol, details of PAT, outcome measurement scale, results (mean), and the details of relapses rate and follow-up.

## Results

### Outcome of study articles

In the initial literature search, from January 2000 to November 2015, 1095 articles were identified from the four databases: Medline (n = 260), Cumulative Index to Nursing and Allied Health Literature (CINHAL) (n = 80), PubMed (n = 346), Scopus (n = 408). In the first stages of the screening, 1050 articles were excluded through screening the title and abstract based on inclusion and exclusion criteria and we included 45 articles for further screening. At the second stages of screening process, we excluded 33 articles due to the following main reasons: full text was not available, full text articles was not in English, conference proceedings, duplication of articles, children over than 2 years of age, neglected clubfoot, clubfoot associated with other problems, survey studies, short-term outcome, other treatments such as operative treatment, Ponseti method with different casting materials, different purpose of the studies other than finding effectiveness of Ponseti method among children with clubfoot. As a result, 12 studies were satisfied our selection criteria for the systematic review study after application of all inclusion and exclusion criteria in 45 articles [49–60]. Among the twelve studies, there were four prospective studies [49–52], one multicenter clinical study [53], one consecutive clinical series [54], two randomized controlled study [55, 56], one cohort study [57] and other three clinical studies [58–60].

### Patient characteristics

The summary of all articles is described in Table 1. There is a total of 852 clubfoot children with 1206 clubfeet were included in this review. In the 12 studies, a total of 293 bilateral clubfoot children are identified [49, 50, 52, 53, 55, 56, 58–60]. Few studies reported that the affected side of the foot is either right side or left side of the foot. [50, 51, 55, 60]. One of the study [54] did not mention the type of clubfoot (affected side). The number of castings, duration of treatment, follow-up is varied from one study to another study. The average initial presentation of casting would determine the effectiveness of the treatment. Therefore, the clubfoot intervention should be started as early as possible [3]. In most of the studies, Ponseti method of treatment was started after immediate birth

**Table 1:** Characteristics of the studies.

References	Study design/ number of children/feet	Types of Intervention/groups/gender	Side of clubfoot (Bilateral & unilateral clubfoot)	Age at Initial casting/ Number of casting	Bracing protocol	PAT /Surgery/	Outcome measurement/ Results (mean)	Relapse/follow up period(Mean)
**Elgohary &** **Abulsaad, 2015**	Prospective study/ 41 Children /66 CF	Traditional Ponseti method (20 Children/ 34 feet/ B = 14; G = 6. Accelerated Ponseti method (21 children/32 feet/B = 12; G = 9.	TPM: 14 children with BCF and 6 children with UCF. APG: 11 BCF and 10 UCF	TPM Group: 10.7 ± 6.28 weeks. APM Group: 11.57 ± 6.9 weeks (from two to twenty six weeks). TPM: One casting per week (4.88 ± 0.88 casting for full correction). APM: Casting twice per week (5.16 ± 0.72 casting for full correction)	Modified Denis–Browne orthosis (70 degree of external rotation on affected foot and 40 degree of external rotation on normal side).	TPM: 91.2% (31 feet of 34) APM: 93.8% (30 feet of 32)	Pirani score: clubfoot with < 4 of Pirani score was included. After the intervention, TPM: 5.17 ± 0.62 (range 4–6) to 0.49 ± 0.42 (0.0–1). APM: 5.13 ± 0.61 (range 4–6) to 0.52 ± 0.38 (0.0–1).	TPM: 14.7% (equinus, heel varus and/or forefoot adduction); APM: 15.6%
**Colburn & Williams, 2003**	34 children (B = 28;G = 6) /57 CF	Ponseti method	BCF– 23 infants; UCF- 11infants	Children with clubfoot were included from first day to 6 months. Average of 4.8 casting (ranges from 3–7); Average of 4.8 casting (from 3 to 14) for those previously treated with other treatments.	Straight-last shoes with a foot abduction bar with 60° - 70° external rotation of the corrected feet/23 hrs per day/3months. Then, 12 hrs/day until 2 years	34 children (54 feet out of 57 feet) were corrected without PMR (95%). Serial casting, Manipulation, PAT: 38 feet (77%). Manipulation and casting: 28% (16 clubfoot) corrected with manipulation and casting only. 5% only was done by PMR	Dimeglio score was 11.2 average; Range from 7–15) for those who are not received any previous clubfoot treatment. The results of the average score was 11.2 (range: 7–17) in patient received some other treatments previously.	Relapses: 6 children. Regained successful correction with manipulation, serial casting and straight-last shoe with foot abduction bar
**Mohammad Hallaj-Moghaddam et al. 2015**	Prospective study / N = 85	Ponseti method (M = 69%, F-31%)	Bilateral: 61.2%. Eighteen percentages- LCF, and 21 percentages of the clubfoot–RCF.	5.7 castings (4 to 8 casting). One casting/wk. Mean age: 8 days at the time of first casting. It ranges from (1–60 days). Average number of casting: 4–8.	Full time Dennis-Browne splint protocol—6 months. Then, Part time Dennis-Browne splint protocol for 3 years	*PAT*: Tenotomy was performed in 76 patients (89.4%). Successful results with plantigrade foot. The child started to walk at the mean age of 12^th^ months. *Complications*: Pain, tenderness after tenotomy for 3 children, one patient had minor infection after tenotomy. BCF had lower outcome than UCF(2.1 ± 1.0 versus 0.63 ± 3.0)	Dimeglio score: Baseline: 16 ± 3.4; after casting—1.6 ± 6.2.	Relapse rate was 27.1% (follow-up period: 5–72 months)/ Follow—up: Every 3–4 months/1-2 years; After that, 6–12 months follow-up was done.
**Pulak & Swamy, 2015**	Prospective study/N = 40/53 feet	Ponseti method	BCF: 14 Children; UCF: 25 children	Initial presentation 6 weeks/35 cases. Average casts for full correction: 4.9. Average duration of casting < 7 wks in > 85% of the patients. Some cases, it increased up to 10 weeks of casting.	Orthosis: 23hrs/first 3 months. After that, night time only for 2–4 yrs.	Tenotomy was done in 94.3% of the patients.	1. Pirani score. 2. Goniometry (Initial score:). 48 / good results (90.6%). 3- Acceptable & 2 child got unsuccessful correction.	19.5 months (6–12 months). 2 relapses
**Sud et al. 2008**	Prospective randomized study (N = 45/67 CF)	Ponseti method (N = 36) Kite’s Method (N = 31)	Ponseti method: BCF– 13. Kite’s Method: BCF-9. UCF: 23 Patients (9-RUCF;14-LUCF).	< 3 months of age (5–90 days). Ponseti method: 5 to 90 days Kite’s Method: 5 to 90 days. Casting: Ponseti method: 3 to 12 casting. Kite’s Method 3 to 23 casting	Foot abduction bar: 2–3 months (full time). Then, 2–4 years (night only)	Ponseti method: 2 patients Kite’s Method: 10 patients	Dimeglio score: PMG: Baseline 14.39 (SD 3.2), Post intervention: 5 to 19 () KMG: 16.19 (SD 2.8) Post intervention: 9 to 19 (16.19±2.8). Ponseti method: 91.7% full correction. Kite Method: Achieved 67.7% of full correction.	Ponseti group: 7 relapses (21.1%) / 27.24 months; Kite’s method: 8 relapses/ 24.8 months
**Sætersdal et al. 2012**	Multicenter clinical study/ (N = 116/162 CF)	Ponseti method Boys: 72% Girls: 28%	BCF: 46 UCF: 70	First cast: with second day and Ranged from 0–9 days of life. 4 patients (First casting): 18, 37, 58, and 60 days of life. Average casting: 7.2 (3–13).	Standard bilateral foot abduction brace: 63%, Unilateral above-the-knee brace- 32%. Bilateral FAB with soft cast: 3%. No brace: one child	PAT: 79% Soft tissue release: 3%	Pirani Score: 4.8 (2.5–6) ROM of foot and ankle	Relapse: 27 feet Second time casting: 15 feet; Second time tenotomy- 18 PMR: 3 feet; Posterior release: 2. One TA tendon transfer.
**Selmani, 2012**	N = 100 /150 CF	Ponseti method (N = 76); B: 38; G: 20 Kite’s Method (N = 74); B:28 G: 20	PMG: 26- BCF KMG: 24 BCF	after birth PMG: 2–90 KMG: 2–90. Since Birth/ Average casting: PMG—4 to 12 (7.1±1.8); KMG: 4 to 22 (11.34±6.3). Casting 7 to 10 days.	PMG: Dennis—Browne bar Dennis Browne bar splints with open-toe tarsopronator shoes (70° of external Rotation. Protocol: Full time- Until walking age. KMG: Full time night splint, night time only splint. For 4 years- open toe box shoes, straight medial border, lateral flaring of the sole, and reverse Thomas heels.	PAT	Pirani Score: PMG—5.2±0.8. Correction: Achieved 96% (73 Feet (96ft). KMG: Pirani score: ROM: 8.21° - dorsiflexion to 13.32° - plantar flexion.	Relapses: 10 feet (13.7%)/36.2 months in Ponseti group (First year follow-up) Follow-up: Ponseti - 36.2 months ± SD 3.2; Final follow-up: No relapses. Kite group: 35.1 months ± SD 2.5 (33 to 38 months) and 100 feet.
**Morcuende et al. 2004**	Consecutive case series (N = 157/ 256 CF)	Ponseti method (N = 76); B: 107 (68%); G: 50	NA	128 (81%) < 6 months. Lesser than 5 casting (90% of the patients). Full correction: 20 days (range: 14–24 days).	Foot-abduction brace: 2 to 3 months (full-time). Naptime and night for 3 to 4 years	Extensive corrective surgery—4 (2.5%); PAT- 86%	Walking: age of 13 months Complications: 12 patients (8%)—erythema, slight swelling on toes. ROM: Aorsiflexion posttenotomy was 20° (Range 0–35 degrees).	Relapses: 17 (10%) / 26 months (6 months—8 years)
**Pavone et al. 2013**	N = 82/114 CF	Ponseti method N = B: 56(68.29%);	BCF: 32 (39%) UCF: 50 (60.9%)–RCF: 28 (56%) in the UCF and 22 had LCF	Age: 0–36 weeks. Initial casting: 14 days (3–81 days)/ 76 (92.68%) Patients: range 0–12 wks); 4 patients: range from 13–24 wks; 2 patients: 25–36 wks. /6.6 casting (Average)	Denis Browne splint: 24/day for 3 months. Night time: 3 years.	PAT: 82.93% (68 patients)– 28 BCF; 40 UCF.	Pirani score: 5.56 points/ range 4.3 to 6 points. i.e 53 children: 6 points of pirani score; 22 children - Functional Ponseti Scores: 96. 34% (79 patients)–Good or excellent. Complications: phlebostatic Syndrome (2 children). Plaster sore on the talar head side. 2 children: minor heel sores due to D-B.	Relapse: 3 (5 feet) patients (3.7%): one adductus and varus, one equinus, all deformities in one. Follow-up: 4 years (13–83 months)
**Rijal et al. 2010**	N = 38/60 CF	Ponseti method (N = 30); Kite Method (N = 30)	BCF: 22 UCF:16	Less than 2 years of age	Abduction splint with shoes- 3 months: 23 hours/day; then, night time: 2–4 years. Walking: Custom made clubfoot shoes.	PAT: 29 (96%) out of 30 patients.	Hindfoot, mid foot and total Pirani scores (1–10 weeks). Pirani score improved faster in 12 bilateral clubfeet (*P*<0.05). BCF (12 Bilateral clubfoot). Hind foot score ↓ more fastly in PMG (0.7) than KMG (1.31) at 10^th^ weeks. Midfoot score ↓ more fastly in PMG (0.5) than KMG (1.04) at 10^th^ weeks. Total score ↓ more fastly in PMG (1.2) than KMG (2.36) at 10^th^ weeks. UCF (12 Patients): PMG had significant improvement at 8^th^ week	Weekly follow-up/10 weeks
**Sanghvi & Mittal, 2009**	Randomized study/ N = 42 /64 CF	Ponseti method (N = 21); B:13; G:8. 30 clubfeet Kite Method: N = 21; B:14; G:7. (34 clubfeet)	KMG: 13 BCF, RCF- 5, LCF-3. PMG: 9 BCF, 6 RCF, 6 LCF	0–36 weeks. Number of casting: PMG: 7; KMG:10. Duration of treatment for full correction: PMG: 10 wks; KMG:13 wks	KMG: full-time splinting; After walking age: night time only splint and daytime–shoes (4–5 years) with an open toe box, lateral flaring of the sole, straight medial border, and reverse Thomas heels shoes. PMG: open-toe tarsopronator with D-B bar (70° of external rotation); UCF: 40° to 45° of external rotation of normal foot. Full time and then night time only and shoes for 4–5 years.	PMR: 3 Patients in KMG	Kite method: 79% Ponseti method: 87%. ROM: 12 degrees of dorsiflexion at PMG and 6° at KMG.	Follow-up: 3 years). 3 Relapses in KMG. One bilateral relapses in PMG.
**Segev et al. 2005**	Cohort study/ 72 infants	Ponseti method (N = 32); 48 CF, B: 20, G: 12. Traditional method (Modified Kite and lovell technique): N = 40 (61 feet)/B:31;G:9	PMG: 50% BCF and left are more involved. TM: 21 BCF	Above knee casting. PMG: 96% patients were treated from the birth. One patient: 3 wks and another patient: 6 wks. TM: duration of casting: 4.0 months (Average).	PMG: Dennis Brown splint for 3 months/24hrs, then night time only until 2 years.	PAT: 47 CF in PMG (Average 2.4 age months); TM: 29 PMR and 6 PR.	Dimeglio-Bensahel scoring. Before the treatment of PMG: 11.9 and after 3.2 (average). 94% achieved in PMG.	Follow-up: 54.9 months (44–68 months) in TM; PMG– 29.2 months. 3 residual deformities. 44% residual deformity at TM

PMR,Posteromedial release; CF,clubfoot; TPM,Traditional Ponseti method; TM, Traditional method; PMG, Ponseti method group; KMG, Kite method group, UCF,Unilateral clubfoot; BCF, Bilateral clubfoot; RCF,Right side clubfoot; LCF, Left side clubfoot; ROM,Range of motion; wks, weeks; PAT,Percutaneous Achilles tenotomy.

## Discussion

In the selected twelve studies, most of the studies have compared the effectiveness of the Ponseti method with the Kite method. There are five studies used Kite method among the twelve selected studies [50, 55–57, 59]. One of the study used accelerated Ponseti method to compare the current existing method of intervention for the clubfoot deformity, and the results of this study stated that the accelerated Ponseti method is safe as a traditional Ponseti method of treatment for clubfoot intervention [49]. The Ponseti treatment regime included manipulation, serial casting, Achilles tendon tenotomy and Bracing—Foot abduction brace [7, 19, 20].

### Casting techniques and numbers of casts

In this review, we assessed the number of castings used in the studies, and all of the selected 12 studies reported the number of casts used to achieve the full correction of clubfoot (35–46). Five studies [50, 55–57, 59] used Kite method techniques to compare with the Ponseti method in the correction of clubfoot. These studies reported that Ponseti method achieved the initial correction in shorter time and used fewer casts than the Kite method. The percentages of Ponseti method’s correction success rate was 96% (follow-up time- 36.2 months) and the Kite’s method full correction success rate was 74.3% at the time of an average of 35.1 months [59]; Another study by Sud et al.2008, achieved 91.7% in Ponseti method (Average of 27.24 follow-up) and Kite method 67.7% at time of 24.8 months follow-up [50]. Although the achievement of correction rate was similar in each group (Ponseti-87% & Kite method-79%), the average of casting was less in Ponseti group (7 casts in Ponseti and 10 casts in Kite’s method) and the duration of treatment took only 10 weeks in the Ponseti method and 13 weeks for Kite method [55]. In Elgohary and Abulsaad (49) study, an average of 4.88 ± 0.88 castings (4–7 casts) were used to achieve the full correction of the clubfoot for traditional Ponseti method group and the average of 5.16 ± 0.72 cast (4–7 casts) in the accelerated Ponseti group, in which 84.8% feet required less than 5 casts in both groups. Another study [59]reported that an average 4–12 casting was only used to get the full correction of clubfoot, but, in the Kite method took an average of 4–20 castings; and also between the casting duration periods varied in their study (7 to 10 days per casting).

On the other hand, the accelerated group achieved full correction of clubfoot from 11 to 12 days (castings twice per week) and for the traditional ponseti method group was 21–42 days. Also, the accelerated group achieved nearly same as a traditional ponseti group in a short period of time [49]. However, in the accelerated Ponseti technique, the casting was performed two times a week. Previously, Morcuende et al. 2005 studies tried the accelerated Ponseti techniques with every 5 days casting instead of 7 days once, and Xu [61] studied with casting twice a week, and the results of both studies showed successful correction as same as traditional method and also the duration of the treatment period was less than the original method. Of these 12 selected studies, one study achieved the full clubfoot correction with less than 5 casts (average of 4.9 casts) in 75% of the cases [52]. Only one study described about the casting procedure time, and it took 5 to 9 minutes for each casting (Average 4–8 casting) and for Achilles tenotomy with last casting ranged from 6 to 11 minutes [51]. In Morcuende, Abbasi (18) study, the following steps of casting procedures were performed to maintain the correction of clubfoot: in the first step casting was performed from toe to lower knee and in the second step casting was performed from the knee to thigh area, and 90% of patients were treated with less than 5 casts. Among the selected studies, one of the study [50] stated that the casting treatment is considered as failure if not achieved full correction within a year, and these patients referred to surgery after obtaining the consent from the patient. In this study, Ponseti method was compared with Kite method, in which the Ponseti method took less casting ranged from 3–12 for achieving full correction of the clubfoot. At the same time, 3–23 casts were used in the Kite method. According to the Pavone, Testa (60) more casting is required if the initial deformity is severe or initial treatment is started after 15 weeks of birth. However, there is a still controversy in the impact of late presentation of treatment and outcome of the results. Some authors reported that late presentation of treatment does not affect the outcome of the results [54, 62–65]. At the same time, the study of Abdelgawad et al. stated that 6.6% failure rate of Ponseti treatment due to the late presentation of the treatment. But one of the selected studies for this review, Pavone, Testa (60) reported that there is no correlation between age of presentation and final outcome of range of motion of the feet even though taking more casts, and the clubfoot was corrected with the range of 5–10 casts in their study. Only two of the studies [56, 59] from this review conducted post casting checking such as margin of the cast, neurovascular assessments, as well as the patients were instructed to see other complications: swelling, discoloration of the skin of the toes and baby’s excessive crying. Out of 12 selected studies, only one study [53] treated the clubfoot children with two different types of casting materials, in which 78% clubfoot children were treated with semi-rigid fibreglass cast and 22% of them were plaster of Paris. However, the results of these casting materials are still controversial based on the report of [53]. In addition, the average of 7.2 casts (total of 162 feet) was used in this study to achieve full initial correction including the final cast after tenotomy. Only 3–5 casts were used for 4 children those had a mild foot deformity. Among 162 feet treated in this study, 15 feet (10 bilateral brace & 5 unilateral brace) were treated with recasting and also this study reported that those who are treated with unilateral brace had more casts than bilateral brace. At the same time, the corrected clubfoot should be maintained by foot abduction braces to prevent the relapses or recurrences of clubfoot [30, 64, 66–70].

### Bracing

Foot abduction braces (FAB) of the Ponseti method protocol are essential to maintain the corrected clubfoot and to avoid the relapses. In these 12 studies, we noticed variations in the bracing protocol schedule and it ranges from 2–6 months for full time or 23 hours, and then followed by 2–5 years in night time [49–60]. Several studies reported that braces need to wear for full time or 24 hours per day up to 2–3 months [51, 54, 60].]. All these moreover, Hallaj-Moghaddam, Moradi (51) study reported that they used full time abduction brace up to 6 months. Three studies stated that full time abduction braces need to wear 23 hours per day up to 2–3 months [52, 56, 58]. Two of the authors from this review reported that the children (clubfoot) used the splint up to the walking age, and then worn the braces night time for up to four years [55, 59]. However, originally the Ponseti method suggested wearing the full time brace up to two to three months and thereafter at night time for up to 3–4 years. One study [53] used different types of braces and changed their brace type in the bracing protocol: 62% of children were used FAB, then 30% user changed into Mitchell from Markell, and 12% user changed into flexible custom-made unilateral above-the-knee brace from bilateral FAB, and 3% are changed into a softcast or scotchcast removable brace/cast. Moreover, we observed that a change in the position of correcting foot varies in the bracing regime. For example, one of the study used 70 degrees of external rotation of clubfoot [49]; another study positioned the feet in a 60–70 degrees of external rotation in their bracing protocol. Non-compliance brace causes the relapse of correcting clubfoot, which affects the success of the Ponseti treatment [71].

### Relapses and its characteristics

Several studies reported that Ponseti method is a successful conservative method to correct the clubfoot deformity [57, 72, 73]. However, previous literatures reported that 10–30 percentages of the relapses are very common [74, 75]. In this review, of the 12 studies, we found that relapses in nine studies [49–52, 54, 57–60, 62]. The maximum of relapse rate was 27.1% [51] and the lowest relapse rate (two relapses) was observed in Pulak and Swamy [52]study. From the selected 12 articles, one of the study [51], stated that maximum relapses rate (27.1%) at the end of the follow-up (average of 5–72 months) period. Total of 76 patients (89.4%) underwent the tenotomy surgical procedures in this study. Few studies reported the relapse pattern of the clubfoot after the initial correction such as fore foot adductus, hindfoot varus, midfoot cavus and ankle equinus [49, 51, 57, 59]. For example, Pavone et.al. [60] reported in their study about 3% of relapses rate, one foot was relapsed by adductus and varus relapse pattern, another foot was affected by equinus, and one foot was totally relapsed with all four components of clubfoot. These relapses occur due to non-compliance with Denis browne splint, and infrequently use of splint because of lack of education and socioeconomic status of parents of clubfoot children [60]. But, in few studies, clubfoot relapse pattern is not clearly described [53, 58]. We observed in Morcuende et.al. study, 11% of the clubfoot children had relapses, and relapses are corrected by manipulation, re-casting, and tendoachilles tenotomy. However, 2.5% of the relapses patients were treated by tibialis tendon transfer and lengthening of Achilles tendon. In Elgohary and Abulsaad (49) study, 14.7% of clubfeet had relapsed due to non-compliance of brace, with 90% of relapses cases had initial high Pirani score (90%). At the same time, Dobbs et al. reported that relapses are not dependent on the initial severity of the clubfoot. Other studies, relapses: 6 cases [58], 27.1% [51], 21.7%[50], 27 feet [53], 13.2% [59], and 2 cases [52] were noticed in the selected articles for our review.

### Relapsing factors

Ponseti practitioners recommended that clubfoot should be treated as early as possible after birth to avoid the relapses and to achieve the full correction of clubfoot [7,16, 26, 28, 59]. Especially, to avoid the recurrence or relapse pattern of the clubfoot, compliance of foot abduction braces is necessary in the Ponseti treatment [30, 54]. The relapses of clubfoot are evaluated by either foot morphology or Dimeglio score or Pirani scoring system [72–75]. It can also be evaluated or classified as minor or major relapses based on how much necessity of surgical release requirement [67].

Approximately 78% of the clubfoot relapses occur due to the reason of non-compliance foot abduction braces, and 7% of relapses occur with compliance of foot abduction braces [72]. Non-compliance of foot abduction braces is having a tendency to induce the relapse of the patient [30, 54]. Relapses are not associated at age of presentation, numbers of casting, and previous history of unsuccessful treatment. It is observed that the noncompliance bracing is being the main issue for relapses for the clubfoot and bracing protocol was not strictly followed by caregivers or family members [70]. Although non adherence of bracing is the leading cause of relapse, number of researches suggested other different causative or risk factors for relapses of correcting clubfoot such as low educational level of parents, low income (< US $20,000), and Native American ethnicity [71]. In our selected review articles, Morcuende et al. study [54] states that noncompliance is 17 times greater than compliance of braces for occurrence of relapses and their study referred 2.5 patients (Children with non-compliance of FAB) for anterior tibial tendon transfer to the third cuneiform surgery to avoid additional relapses. Poor socio-economic status and lack of understanding causes relapses in two patients [60]. Three studies [52,55–56] do not describe the causes of relapses in their studies. Three studies were only described the compliance of braces [58– 60]. Some authors suggested that poor experience in casting techniques, improper tenotomy surgeries, ill-fitting of FAB as well as poor education and socioeconomic status of parents for the reason of recurrence [54,60,76], short term follow-up [57]. One selected article in our study reported that 13.7 percentages of relapses occurred in the first year of age in follow-up [59] and so that they are drawn a conclusion relapses is not related to age factor or severity of the foot [50,59]. Of twelve study, only one study [51] was tried to analyze the relapsing related to different factors such as gender, age, age at first casting, clubfoot with other deformities, walking age, number of castings, initial severity score (Dimeglio score), tenotomy, and satisfaction. However, the brace compliance was 97 percentages but the relapse occurred in 27% of the patients and this study suggested that relapse rate might be due to noncompliance of braces, initial severity, below knee casting, and low education level of level of parent’s. Previous literatures have only been discussed about the relationship between the compliance of braces and relapses. According to the Morcuende et al. “Compliance” defined as the children not wearing the foot abduction orthosis for a period of 10 hours per day consider as compliance [54] and Dobbs et al. defined as “complete discontinuation of FAB” [30]. However, there were no studies described about the grade of compliance of braces in details in our selected studies except Sætersdal et al. study [53]. In our selected study for this review[53] graded the compliance of brace as Excellent: use the braces continually until the follow up (4 years of age) or at least 10 hours every day for night time regime; Good: use the braces continually until the follow up (2 years of age) or at least 6 to 8 hours every day for night time regime; Fair: If the braces used less than 2 years of age or less than 6 hours; Non-compliant: If the braces discounted before the age of one year. However, there is no studies described the any grade of compliance for relapses in our selected studies. Identification of relapse pattern, and adherence to the original protocol of the Ponseti method with understanding of detailed manipulation, casting, bracing is necessary to prevent the relapses [3, 19, 38].

### Evaluation of treatment outcome and surgical recommendations

In the evaluation or outcome of the clubfoot treatment by Ponseti method, most of the studies, using Pirani scoring system or Dimeglio scoring system as outcome measurement scale for assessing the clubfoot deformity. In addition, these measurement scales are also helps to predict whether the percutaneous Achilles tenotomy is needed or not to correct the equinus [77–78]. If the initial score of Pirani is greater than 5, then it should be corrected with Achilles tenotomy to correct the equinus but the score is less than 3, there is no requirement of this surgery [78]. On the other hand, Morcuende et al.[54], used initial correction of the clubfoot, the rate of extensive corrective surgery, and relapses rate as an outcome measures to evaluate the effectiveness of intervention. The results of Morcuende et al. study [54] showed are: Average achievement of dorsiflexion at ankle is 20 degrees, and initial correction was achieved in 20 days, and relapses were noticed about 10% followed by initial treatment. Some studies, used goniometry to assess the ankle range of motion (ROM) such as dorsiflexion and planter flexion to find out the effectiveness [52] of clubfoot deformities. The results and measurement outcomes of the selected 12 studies are described in the Table 1. The Dimeglio score was reduced from 16 ± 3.4 to 1.6 ± 6.2 at the end of the follow-up [51]; and was an average of 11.2 (received no previous treatment and received previous treatment) before the casting at Colburn & Williams study. However, the Colburn & Williams study does not explain how much the Dimeglio score reduced after casting and follow-up, and this study stated that performed posteromedial release surgery for 3 children (received previous treatment group) [58]. Some of the studies used Pirani scoring system for evaluation but in the outcome evaluation the study did not clearly described the pre and post or follow-up of Pirani scoring outcome [59]. In Pavone et al. study [60], the Pirani score was an average of 5.56 before the casting treatment, and thereafter the results suggested that 82.93% for percutaneous tenotomy after the casting. However, this study also not described about the post Pirani scores but in the follow-up (mean of 4 years follow-up), 98.78% patients had normal passive range of motion, achieved good to excellent results (96.34%) in the functional Ponseti scoring system score [60]. Some studies reported the all parts of the Pirani score including total score (5.6) and the hindfoot score and midfoot score was at 2.9 and 2.8 respectively, and 94.3% of the cases were referred to tenotomy [52], and in another study the hindfoot Pirani score reduced from 2.62 to 0.7 in Ponseti method and 2.79 to 1.31 Kite's method at the 10^th^ weeks of follow-up. Also, the midfoot score of Ponseti method and Kite method’s decreased from 2.62 and 2.7 to 0.5 and 1.04 respectively, and 96% went for tenotomy surgery in the Ponseti method [56]. One study [53] reported the range of motion as well as Pirani score, the mean Pirani score was 4.8 before the treatment. Thereafter, Pirani score of 0 or 0.5 in 78% of the patients, 1 or 1.5 in 22% of the patients, 3.5 score was noted only one patients. In addition, we observed in their study is about 92% achieved more than 15 degrees of dorsiflexion, 84% of feet freed from adduction deformities, and 93% had more than 40 degrees of external rotation, and 4% feet had adducted deformity. Only one study [55] used the following measurements method to assess the outcome: a) Clinical assessments (relapses, passive ROM, appearance of the foot, muscle power, calf muscle atrophy, size of the foot size, and other complications); b) Functional assessments (Gait pattern, functional limitation, pain, satisfaction of the patients and shoe comfort wear); c) Radiological assessments- talocalcaneal angle, talo first metatarsal angle, and talocalcaneal index. All these three components together graded as excellent-85 to 100 points; good -70 to 84 points); fair—60 to 69 points; poor <60 points. The overall results of this study showed 87% success rate in Ponseti method and 79% in Kite method. In Segev study, 94% of the feet corrected successfully in the Ponseti method (the average of Dimeglio score reduced from 11.9 to 3.2), and tenotomy was performed in 47 feet. At the same time, 6% had residual deformity [57].

### Strength and limitations of this systematic review

The main strength of this review is that it thoroughly followed a systematic method and analysis method than previous published few systematic review articles and several reviews on clubfoot with Ponseti method. Also, this review study found that 2 or 3 articles selected in this review, even though they compared the Ponseti method with Kite method and showed that Ponseti techniques are superior to the Kite method, the publications were same as other ones without changing the written script (protocol, some of the results and discussion parts of the articles), and other details.

## Conclusion

In conclusion, this study reviewed all aspects of Ponseti techniques, comparison of the Ponseti method with the Kite method, and the outcome of the results, number of casts used in clubfoot intervention, number of patients underwent for surgical procedures, and the relapses pattern of clubfoot followed by correction of clubfoot. Overall, this review found that the Ponseti method required fewer casts, shorter duration to achieve the correction, less relapses rate than other methods. On the other hand, few studies were only described the relapse pattern, and causes of relapse. There is still lack of information regarding the causes of relapse or recurrences of clubfoot. Some of the studies reported that poor socioeconomic status and bracing compliance. However, the authors did not describe the duration of discontinuity of braces or relapses pattern, or any grading for bracing compliance in their outcome. Also, we noticed that two or three studies described the relapse pattern, bracing protocol and discussion of the results, in which they followed previous articles without changing any sentences or paragraph of the previous articles. It seems that there is a need for more systematic or Cochrane review in this area for doing careful evaluation of clubfoot intervention and also to avoid the duplication of the previous articles, and to evaluate and review the relapse pattern, adherence of bracing protocol and long term follow-up. This systematic review study has some limitations such as this study reviewed only children with less than two years old. Therefore, this study does not include clubfoot children with more than two years old, neglected clubfoot with using the Ponseti method. In the future study, it is recommended that to do more literature search on databases to review the neglected clubfoot, clubfoot with other abnormalities, more randomized control studies with using Ponseti method and other interventions.

## Supporting information

S1 File Electronic search strategy. (PDF)

S2 File The PRISMA checklist. (DOC)
