# Supplementary material for: Ponseti method in the management of clubfoot under 2 years of age: A systematic review
Source: PLoS One. 2017 Jun 20;12(6):e0178299. doi: 10.1371/journal.pone.0178299 (PMC5478104; doi:10.1371/journal.pone.0178299)
Supplement: S1 File — (PDF) [file pone.0178299.s001.pdf]

Electronic search strategy

Please provide the full electronic search strategy for at least one database, including any limits used, such that it could be repeated.

I have enclosed the file how database searched to select the articles.
Steps 1. Used key words “clubfoot” and “Ponseti Method”. or “Congenital talipes equinovarus” .
Steps 2: Limit the year (2000 to 2015).
The details are given below on the next page as an example

PubMed ▼ (CLUBFOOT) AND PONSETI METHOD

Format: Summary **Sort by**: Most Recent **Per page**: 20

Search results

Items: 1 to 20 of 346

Filters activated: Publication date from 2000/01/01 to 2015/11/31. Clear all to show 415 items.

1. Treatment of persistent forefoot adduction during **ponseti method** in treatment of idiopathic talipes equinovarus by minimal soft release.
   Abdullah el SA.
   J Orthop. 2015 Jun 6;13(3):230-4. doi: 10.1016/j.jor.2015.05.003. eCollection 2016 Sep.
   PMID: 27408483

2. A review of existing trauma and musculoskeletal impairment (TMSI) care capacity in East, Central, and Southern Africa.
   Chokotho L, Jacobsen KH, Burgess D, Labib M, Le G, Peter N, Lavy CB, Pandit H.
   Injury. 2016 Sep;47(9):1990-5. doi: 10.1016/j.injury.2015.10.036. Epub 2015 Oct 26.
   PMID: 27178767

3. Treatment of **Clubfoot** With **Ponseti Method** Using Semirigid Synthetic Softcast.
   Aydin BK, Sofu H, Senaran H, Erkocak OF, Acar MA, Kirac Y.
   Medicine (Baltimore). 2015 Nov;94(47):e2072. doi: 10.1097/MD.0000000000002072.
   PMID: 26632713

4. Treatment of Severe Recurrent **Clubfoot**.
   Radler C, Mindler GT.
   Foot Ankle Clin. 2015 Dec;20(4):563-86. doi: 10.1016/j.fcl.2015.07.002. Epub 2015 Sep 14. Review.
   PMID: 26589079

5. Treatment of Idiopathic **Clubfoot** in the **Ponseti** Era and Beyond.
   Chu A, Lehman WB.
   Foot Ankle Clin. 2015 Dec;20(4):555-62. doi: 10.1016/j.fcl.2015.08.002. Epub 2015 Oct 23. Review.
   PMID: 26589078

6. Factors affecting adherence with foot abduction orthosis following **Ponseti method**.
   Göksan SB, Bilgili F, Eren İ, Bursalı A, Koç E.
   Acta Orthop Traumatol Turc. 2015;49(6):620-6. doi: 10.3944/AOTT.2015.14.0348.
   PMID: 26511688

7. CORR Insights®: Does Strict Adherence to the **Ponseti Method** Improve Isolated **Clubfoot** Treatment Outcomes? A Two-institution Review.
   Nogueira MP.
   Clin Orthop Relat Res. 2015 Oct 20. [Epub ahead of print] No abstract available.
   PMID: 26487044

8. Congenital idiopathic talipes equinovarus before and after walking age: observations and strategy of treatment from a series of 88 cases.

Faldini C, Traina F, Nanni M, Sanzarello I, Borghi R, Perna F.
J Orthop Traumatol. 2016 Mar;17(1):81-7. doi: 10.1007/s10195-015-0377-4. Epub 2015 Sep 26.
PMID: 26409466

9. Does Strict Adherence to the **Ponseti Method** Improve Isolated **Clubfoot** Treatment Outcomes? A Two-institution Review.
Miller NH, Carry PM, Mark BJ, Engelman GH, Georgopoulos G, Graham S, Dobbs MB.
Clin Orthop Relat Res. 2016 Jan;474(1):237-43. doi: 10.1007/s11999-015-4559-4. Epub 2015 Sep 22.
PMID: 26394639

10. The Heel Pad in Congenital Idiopathic **Clubfoot**: Implications of Empty Heel for Clinical Severity Assessment.
Adegbehingbe OO, Asuquo JE, Joseph MO, Alzahrani M, Morcuende JA.
Iowa Orthop J. 2015;35:169-74.
PMID: 26361461

11. Photovoice and **Clubfoot**: Using a Participatory Research **Method** to Study Caregiver Adherence to the **Ponseti Method** in Perú.
Pletch A, Morcuende J, Barriga H, Segura J, Salas A.
Iowa Orthop J. 2015;35:160-8.
PMID: 26361460

12. Congenital **Clubfoot**: Early Recognition and Conservative Management for Preventing Late Disabilities.
Liu Y, Zhao D, Zhao L, Li H, Yang X.
Indian J Pediatr. 2016 Nov;83(11):1266-1274. Epub 2015 Sep 5. Review.
PMID: 26341712

13. Outcome of Percutaneous Tenotomy in the Management of Congenital Talipes Equino Varus by **Ponseti Method**.
Alam MT, Akber EB, Alam QS, Reza MS, Mahboob AH, Salam SI, Islam MS, Ara I.
Mymensingh Med J. 2015 Jul;24(3):467-70.
PMID: 26329941

14. Trauma and orthopaedic capacity of 267 hospitals in east central and southern Africa.
Chokotho L, Jacobsen KH, Burgess D, Labib M, Le G, Lavy CB, Pandit H.
Lancet. 2015 Apr 27;385 Suppl 2:S17. doi: 10.1016/S0140-6736(15)60812-1. Epub 2015 Apr 26.
PMID: 26313063

15. Kite versus **Ponseti Method** in the Treatment of 235 Feet With Idiopathic **Clubfoot**: Results of a Single Romanian Medical Center.
Derzsi Z, Nagy Ö, Gozar H, Gurzu S, Pop TS.
Medicine (Baltimore). 2015 Aug;94(33):e1379. doi: 10.1097/MD.0000000000001379.
PMID: 26287427

16. Lateral Tibiocalcaneal Angle As a Determinant for Percutaneous Achilles Tenotomy for Idiopathic Clubfeet.
Kang S, Park SS.
J Bone Joint Surg Am. 2015 Aug 5;97(15):1246-54. doi: 10.2106/JBJS.O.00076.
PMID: 26246259

17. [Arthrogryposis: clinical manifestations and management].
Ayadi K, Trigui M, Abid A, Cheniour A, Zribi M, Keskes H.
Arch Pediatr. 2015 Aug;22(8):830-9. doi: 10.1016/j.arcped.2015.05.014. Epub 2015 Jul 2. French.
PMID: 26141802

18. [Steenbeek Brace: Patterns of Wear.]
Agarwal A, Shaharyar A, Kumar A, Bhat MS, Mishra M.
Foot Ankle Spec. 2016 Feb;9(1):13-6. doi: 10.1177/1938640015592838. Epub 2015 Jun 29.
PMID: 26123547

19. Mid-term results of a physiotherapist-led **Ponseti** service for the management of non-idiopathic and idiopathic **clubfoot**.
Dunkley M, Gelfer Y, Jackson D, Parnell E, Armstong J, Rafter C, Eastwood DM.
J Child Orthop. 2015 Jun;9(3):183-9. doi: 10.1007/s11832-015-0658-8. Epub 2015 Jun 14.
PMID: 26072327

20. Difficulties in maintenance of **clubfoot** abduction brace and solutions - maintenance of **clubfoot** abduction brace, locks and keys.
Memon I, Bhatti A, Ali P, Mahmood K; M. Saeed Minhas..
J Pak Med Assoc. 2014 Dec;64(12 Suppl 2):S70-5.
PMID: 25989785

Back to top
